# Standardized Comparison of Voice-Based Information and Documentation Systems to Established Systems in Intensive Care: Crossover Study

**DOI:** 10.2196/44773

**Published:** 2023-11-28

**Authors:** Arne Peine, Maike Gronholz, Katharina Seidl-Rathkopf, Thomas Wolfram, Ahmed Hallawa, Annika Reitz, Leo Anthony Celi, Gernot Marx, Lukas Martin

**Affiliations:** 1 Department of Intensive Care Medicine and Intermediate Care University Hospital RWTH Aachen Aachen Germany; 2 Clinomic Group GmbH Aachen Germany; 3 Laboratory of Computational Physiology Harvard–MIT Division of Health Sciences Technology Cambridge, MA United States; 4 Beth Israel Deaconess Medical Center Boston, MA United States

**Keywords:** artificial intelligence, documentation, ICU, intensive care medicine, speech-recognition, user perception, workload

## Abstract

**Background:**

The medical teams in intensive care units (ICUs) spend increasing amounts of time at computer systems for data processing, input, and interpretation purposes. As each patient creates about 1000 data points per hour, the available information is abundant, making the interpretation difficult and time-consuming. This data flood leads to a decrease in time for evidence-based, patient-centered care. Information systems, such as patient data management systems (PDMSs), are increasingly used at ICUs. However, they often create new challenges arising from the increasing documentation burden.

**Objective:**

New concepts, such as artificial intelligence (AI)–based assistant systems, are hence introduced to the workflow to cope with these challenges. However, there is a lack of standardized, published metrics in order to compare the various data input and management systems in the ICU setting. The objective of this study is to compare established documentation and retrieval processes with newer methods, such as PDMSs and voice information and documentation systems (VIDSs).

**Methods:**

In this crossover study, we compare traditional, paper-based documentation systems with PDMSs and newer AI-based VIDSs in terms of performance (required time), accuracy, mental workload, and user experience in an intensive care setting. Performance is assessed on a set of 6 standardized, typical ICU tasks, ranging from documentation to medical interpretation.

**Results:**

A total of 60 ICU-experienced medical professionals participated in the study. The VIDS showed a statistically significant advantage compared to the other 2 systems. The tasks were completed significantly faster with the VIDS than with the PDMS (1-tailed *t*_59_=12.48; Cohen *d*=1.61; *P*<.001) or paper documentation (*t*_59_=20.41; Cohen *d*=2.63; *P*<.001). Significantly fewer errors were made with VIDS than with the PDMS (*t*_59_=3.45; Cohen *d*=0.45; *P*=.03) and paper-based documentation (*t*_59_=11.2; Cohen *d*=1.45; *P*<.001). The analysis of the mental workload of VIDS and PDMS showed no statistically significant difference (*P*=.06). However, the analysis of subjective user perception showed a statistically significant perceived benefit of the VIDS compared to the PDMS (*P*<.001) and paper documentation (*P*<.001).

**Conclusions:**

The results of this study show that the VIDS reduced error rate, documentation time, and mental workload regarding the set of 6 standardized typical ICU tasks. In conclusion, this indicates that AI-based systems such as the VIDS tested in this study have the potential to reduce this workload and improve evidence-based and safe patient care.

## Introduction

### Overview

Intensive care medicine is the interdisciplinary treatment of patients with critical illnesses in specialized wards called intensive care units (ICUs) [1,2]. Patients admitted to ICUs have complex courses of disease and related treatments. However, ensuring adequate and safe measures in the ICU is often difficult due to a combination of short stays among patients, a high cognitive workload, and a limited number of rotating staff members [3]. The staff’s time distribution is crucial for patient care and treatment. The direct contact between doctors and patients plays a crucial role in patient and family satisfaction as well as physicians’ work satisfaction [4].

Patient contact, including treatment and communication, only makes up about one-fifth of the doctors’ work time, while about one-third of their time is spent on documentation and data interpretation [5]. The documentation for patients with critical illnesses is complex and therefore labor-intensive [2]. This leads to an abundance of information with a high density of data arising from different bedside devices, consequently making their interpretation difficult and time-intensive [3]. Thus, documentation and evaluation tasks make up an increasing part of the physician’s work time and become a major part of work on ICUs [6].

Physicians report that, due to rising requirements in documentation, they are under constant time pressure and complain about lacking time for patient-centered care [7]. This increasing burden is one of the crucial driving forces for burnout syndrome in physicians [8-10]. Similarly, a central part of the nurses’ work on ICUs is the collection and documentation of massive, however critical, amounts of data and information about their patients [11]. This information overload and the redundancy in documentation can impair the ability to recognize the development of critical situations early on [3,12]. In response to this increasing workload, an improved documentation system is needed to enable the ICU staff to focus on their patients and decrease the time spent on documentation.

### Workload in ICUs

Several aspects contribute to the workload in ICUs. First, medical knowledge in general is growing exponentially [13]. Medical guidelines and treatments increase in dynamicity and complexity as medical decisions and treatments at the patient’s bed need to follow the current state of scientific evidence [14]. Additionally, the amount of generated health data doubles every 3 years [15]. Currently, an ICU patient produces more than 1000 data points per hour [15]. Thus, complex patient monitoring often leads to an information overload with unstructured and context-free data [3].

This makes it difficult to extract the most significant (and thus decision-relevant) aspects of a patient’s history and the course of the disease [7,16]. The 2011 study by Ahmed et al [17] proves that the way in which the large amount of data generated in ICUs are presented has an impact on the viewer’s ability to put it into the correct context. The more data are presented to the viewer, the higher the associated error rate. The phenomenon is even aggravated by the introduction of electronic health records and patient data management systems (PDMSs), especially when they present complete, unfiltered data sets [17]. Often, these systems therefore draw the immediate attention of the treating staff, resulting in less focus on the actual patient treatment [18].

These phenomena even have an imminent impact on the staff’s occupational and mental health. It has been demonstrated that the more time physicians spend on less satisfying tasks (such as documentation), the higher the risk for burnout syndrome will become [19]. Shanafelt et al [19] showed in their 2009 analysis that the most important factor for burnout in physicians was to spend less than 20% of their time on the most meaningful activity (odds ratio 2.75, 95% CI 1.13-4.6; *P*<.001). The association between a physician’s subjective work experience and the quality of patient care was already underlined in a 1985 study by Grol et al [20]. A similar finding for nursing staff was made in 2019 by Manomenidis et al [21]. They proved that, connected to burnout syndrome, the hand hygiene compliance in nursing staff was decreasing significantly, leading to reduced patient safety.

### Documentation Systems in ICUs

Documentation facilitates interdisciplinary information flow and enhances continuity in patient care [22]. There are different forms of documentation systems available for this purpose in an ICU setting, such as traditionally used paper-based documentation, electronic health records with PDMSs, and new software developments that make use of speech recognition and artificial intelligence (AI).

Paper documentation has the advantage of being cost-efficient and simple to use, as no IT infrastructure has to be implemented [23]. However, disadvantages include the lack of on-site documentation, the lack of simultaneous access, and redundancy and ineffectiveness in documentation [24]. PDMS solutions have been developed to replace paper files, coordinate records from bedside equipment and laboratories, and thus reduce the ICU team’s workload [25].

Many studies have investigated the pros and cons of PDMS implementation and show heterogeneous outcomes. Some studies underline that electronic health records lead to time savings, uniformity, and readability of the documentation. Ubiquitous and parallel availability of the patient’s files reduces idle time and minimizes interruptions in documentation and data assessment [26,27]. Hence, information flow can be increased while the error rate can be decreased [3]. On the contrary, other studies show that PDMS generates a large amount of data, which, depending on the presentation, can make it difficult to identify relevant data and increase error rates, thus leading to a higher workload [7,17,28,29].

Currently, as many studies have proven, there is an imbalance between the time spent on completing documentation tasks and direct patient care [4,5,7,11,30]. Several studies show that the steady increase in time spent on documentation can be traced back to the introduction of electronic health records [7,25,27,31].

Speech recognition, a technique mainly driven by AI, can support the completion of documentation tasks. It is commonly used in consumer hardware devices and has been proven to increase productivity while reducing costs in the medical domain [28,32]. The use of computerized voice recognition in the medical domain is currently being investigated [33]. Several studies showed a reduction in documentation time with the use of speech recognition [29,33-36]. The reports produced offered greater word variety and more detailed and longer texts [34]. Additionally, an accelerated information flow and an improved subjective efficiency were proven [32,36].

Nevertheless, speech recognition is not well-established; thus, several studies examining the aforementioned technology showed a high error rate with significantly more critical clinical errors [28,35]. The main reasons for the errors in speech recognition are the use of nonnative speakers, difficulties in recognizing medical terms, and the ambient noise common in an ICU setting [33,34].

Speech recognition technologies have the potential to reduce the workload, especially regarding documentation tasks. However, it is necessary to develop a system that addresses the issues currently found in the use of speech recognition in order to establish the technology in clinical settings. This study investigates the use of an AI-based voice recognition technology for typical ICU tasks.

The development and introduction of a new documentation system must be based on the challenges in current technologies. A new system has to be intuitive and easy to understand, especially as the introduction period is often perceived as an addition burden since increased time has to be spent on the same tasks [27,36].

In addition, a new system should autonomously record and summarize patient data [11,37]. Thus, the vast amount of data produced by an ICU patient should be recorded, saved, and organized in the new system automatically, without the work of the medical team, in order to reduce workload. Flohr et al [3] confirm in their study that the automated collection of all patient data—the ability to view it in summarized form, identify trends, and have clear patient lists—can facilitate decisions and reduce workload and error rates [3].

While on the one hand, speech recognition can possibly reduce the documentational burden, on the other hand, AI can enable a well-structured, relevance-oriented patient presentation and clinical decision support [30,38].

Although of high importance for clinical outcomes, limited studies have been performed to compare new AI-based, voice-controlled documentation and information software with established documentation systems for ICUs (eg, paper documentation and classical PDMS computerized input) [32,34]. In this crossover study, we compare performance, mental workload, documentation accuracy, and user experience between methods. The objective of this study is to compare established documentation and retrieval processes with newer methods, such as PDMSs and voice information and documentation systems (VIDSs). This study compares performance, documentation accuracy, mental workload, and user experience associated with 6 tasks typical of the ICU as they are completed using 3 different approaches (paper-based, PDMS, and VIDS).

## Methods

### Study Design

#### Material

The study design includes 3 different ICU documentation tools.

#### An Established PDMS (IntelliSpace Critical Care and Anesthesia, Version J.01.00; Koninklijke Philips N.V.)

IntelliSpace Critical Care and Anesthesia is a clinical documentation and decision support system that includes a flowsheet, calculations engine, clinical advisories, device interfacing, orders, microbiology and pathology results, dietary and nursing orders, order management, infusion management, and numerous other functionalities [39]. We generated a fictitious patient using medical data mimicking a typical intensive care patient, including laboratory values, findings, demographic data, and other clinically relevant aspects. The validity of the data was confirmed by 2 independent trained intensivists.

#### Paper-Based, Conventional Documentation on Patient Curves (MEDLINQ Curve, Version 03.17)

In order to reflect the complexity of an intensive care patient, the participants received paper-based documentation (“patient curve”) of the same fictitious patient. This record included laboratory values, care reports, an anesthesia protocol, microbiology requests, a document concerning the patient’s belongings, and the patient curve. The curve consisted of 4 pages used to document the patient status for 1 day. The curve was completed for the patient until 6 AM, which was the time the participants were asked to complete the tasks. The biggest emphasis was placed on the fact that the paper-based sheet contained the same clinical information as was included in the other arms.

#### VIDS (Mona, Version P1.2; Clinomic GmbH)

**Figure 1:**
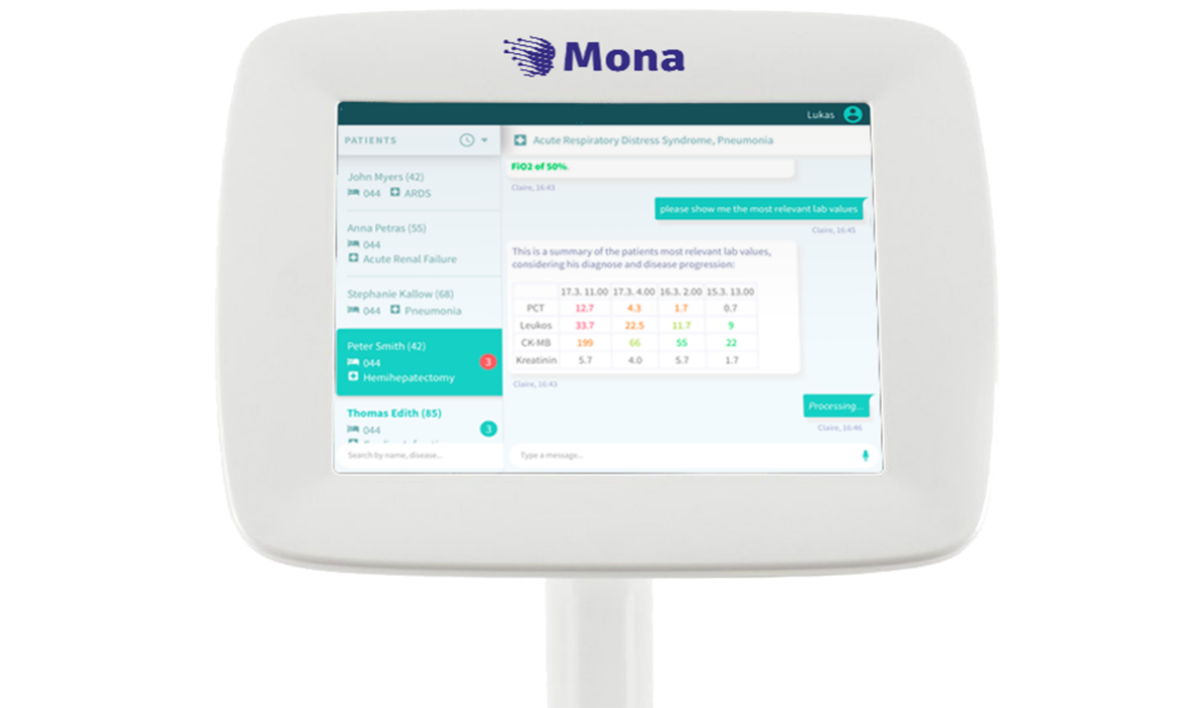
Overview of the voice information and documentation system [40].

In order to equalize potential differences between the study arms as much as possible, the AI-based software was installed on a portable system-on-chip computer (NVIDIA AGX Xavier) so that all study assessments could be performed in the same location. The system was based on adapted, proprietary software (“Mona”). For the purpose of the study, an adapted version of the Mona system was used, containing the following components: (1) voice handling capabilities: natural language understanding and processing; (2) data processing and preparation algorithms; (3) user interface components; and (4) voice synthesis components. The system was running on a Linux-based operation system and was further equipped with a directional microphone (Bose VideoMic NTG microphone) in order to create optimal voice recording circumstances (Figure 1) [40]. The VIDS interacted with an electronic health record for each patient. The system was able to extract and display information from the patient data, enter patient care–related tasks, and navigate through charts. The user activated the system by saying, “Hey Mona.” The system then played a short sound to reflect that voice recognition was activated. Throughout the interaction between the system and the user, the conversation was displayed for the user to read. The system would ask the user for any missing information to complete a task or answer a question. The patient information was shown in the form of tables and abstracts from the flow sheets. After a task was completed or a question was answered, the user had to reactivate the voice recognition with the words “Hey Mona.”

#### Procedure

A total of 60 medical professionals were included in the study. To achieve a sufficient level of significance, the size of the necessary sample was calculated as follows: sample size = [z^2^ × p(1 – p)]/e^2^/1 + [z^2^ × p(1 – p)]/e^2^ × N], where N = population size, z = *z* score, e = margin of error, and p = SD. The assumptions here were as follows: the total number of health care workers in Germany is 5.8 million, of whom 10% are working in ICUs. Confidence level of 0.85, resulting in a *z* score of 1.65 and a margin of error of 0.1. As a result, the required sample size was set at n>52. The definition of “ICU-experienced” included trained physicians, medical students after their fourth study year, and ICU nurses. The participants were recruited between February 5, 2021, and May 14, 2021. Participants were recruited in different domains of ICUs in order to represent the widest possible range of potential users of an information and documentation system. Participation was voluntary and could be terminated at any time without consequences.

First, each participant was informed about all parts of the study and then asked to sign an informed consent form. This was followed by the completion of a questionnaire about demographics, professional background, and technical affinity. The latter was measured using the validated “Fragebogen zur Erfassung der Technikaffinität als Umgang mit und Einstellung zu elektronischen Geräte” (TA-EG, “Questionnaire for the assessment of technology affinity as handling and attitude toward electronic devices”) questionnaire [41] (Multimedia Appendix 1).

**Figure 2:**
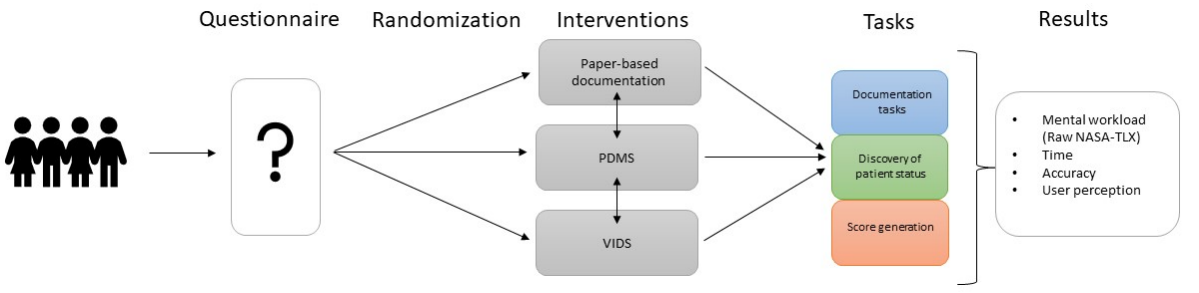
Study procedure. NASA-TLX: National Aeronautics and Space Administration Task Load Index; PDMS: patient data management system; VIDS: voice information and documentation system.

The participants then moved over to the study location. In order to reproduce the high noise level of ICUs during the COVID-19 pandemic, the tests were performed in a noisy simulation environment where other people were simultaneously working and moving around. The participants were asked to work on 6 different tasks typical in ICU workflow. The first 3 of the tasks were documentation tasks, followed by an assessment of a patient’s status (either lactate trend or creatinine levels), and the last one was the generation of the ICU-relevant score “sequential organ failure assessment.” Details on the given tasks are presented in Textbox 1. This list of tasks had to be completed with each of the examined systems mentioned above. Details about the questionnaires and methodology used can be found in Multimedia Appendix 2. The order in which the participants were presented with the different study arms was randomized before the start of the study using the randomize functionality of Excel (Microsoft Corp). The crossover design was chosen to ensure the comparability of all 3 interventions with respect to confounding variables. In addition, it allowed the risk of first- and second-type errors to be kept as low as possible when needing to minimize the number of necessary participants during the COVID-19 pandemic. While the participant executed the tasks, the time needed was measured for each system. Each participant completed the tasks with the respective systems in all study arms. The order of steps in the study can be seen in Figure 2. The detailed solution pathway for each task completion can be seen in Multimedia Appendix 3.

**Tasks presented to the study participants.**

**Documentation**

- “Document 300 mg amiodarone IV now”
- “Document 1.5 g piperacillin/tazobactam (Tazobac) intravenously now/at 10:00 AM”
- “Document the administration of a red blood cell concentrate/fresh frozen plasma at 1:00 AM for procedures numbered 1101002233 and indication active bleeding”
- “Document 20 mg furosemide now”

**Discovery of patient status**

- “What was the lactate trend in the last 12 hours?” Or “how did the creatinine levels develop within the last 4 days?”

**Score generation**

- “What is the patient’s current SOFA score?”

After completing all tasks for 1 study branch, the participants filled out the German translation of the Raw National Aeronautics and Space Administration Task Load Index (NASA-TLX; Raw Task Load Index [RTLX]), a 6-item questionnaire that assesses subjective workload in 6 subscales without using a weighted ranking [42,43]. The shorter version of the NASA-TLX has great use in industry and research due to its simplicity while being equivalent to the task load index, and is thus recommended as a workload assessment tool [44]. For each participant, a mean value was calculated from the answers to the 6 subscales. A high RTLX score correlates with a high mental workload.

Lastly, the participants completed a final questionnaire to assess subjective user perception. This included the meCUE2.0, which is a questionnaire measuring user experience [45]. The meCUE2.0 was filled out for every system (Multimedia Appendix 2). The answers to the 6-point Likert scale were converted into numerical values so that the number 6 represents a maximum positive user experience. Additionally, the net promoter score (NPS) for each system was assessed. This score is a market research metric correlating with actual consumer behavior. It analyzes how likely a company or system is to be recommended by dividing the participants into detractors, neutrals, and promoters [46].

All questionnaires were filled out on a tablet using a publicly accessible, web-based survey system (LimeSurvey, version 3.23.3). Participants’ responses were recorded for each task and subsequently compared against a clinically validated gold standard to assess accuracy. The gold standard was determined by 2 independent ICU specialists. A third independent ICU specialist was consulted in case of disagreement between the 2 physicians to determine the solution for the gold standard. Each error was counted as a negative point. Errors were defined as responses that deviated from the independently generated gold standard or tasks that were not completed.

There was no involvement of patients; all the data used were anonymous and fictitious.

### Statistical Analysis

All statistical preprocessing and analysis were carried out using Excel and SPSS Statistics 27 (version 27.0.0.0; IBM Corp). For all statistical procedures, the α level was set at .05. The results were rounded to 2 decimal digits. According to the central limiting value theorem, an approximative normal distribution was assumed as the sample size was more than 30 [47]. The Levene test was used for the homoscedasticity requirement. A repeated, nonparametric, 1-way ANOVA was used in order to examine potential statistical differences between the different study arms.

### Ethical Considerations

This study was approved by the ethics commission of the Medical Faculty of Rheinisch-Westfaelische Technische Hochschule Aachen (EK370/19).

## Results

### Overview

A total of 60 participants were included in the study; no dropouts occurred during the course of the study. Of the 60 participants, 26 identified as male and 34 as female. The average age was 32.87 (SD 12.46) years. The age ranged from 21 to 63 years. The groups were represented by 43% (26/60) physicians, 40% (24/60) students, and 17% (10/60) ICU nurses. Within their respective professional groups, 32% (19/60) reported 3-5 years of work experience, 27% (16/60) >10 years, and 18% (11/60) 5-10 years. Overall, 17% (10/60) had <1 year of work experience, and 7% (4/60) of participants reported experience of 1-3 years. In total, 6 participants reported having a native language other than German. Overall, 63% (38/60) of the 60 participants worked in a top-level hospital (>500 beds), 25% (15/60) in an upper-level hospital (300-500 beds), and 12% (7/60) in a primary care hospital (up to 300 beds). The technology affinity score was assessed with a mean of 3.70 (SD 0.47). The detailed characteristics of the study can be found in Table 1.

**Table 1:** Study population.

Variable		Value (n=60)
Age (years), mean (SD)		32.87 (12.46)
**Gender, n (%)**		
	Female	34 (57)
	Male	26 (43)
	Diverse	0 (0)
**Work setting, n (%)**		
	Top-level hospital (>500 beds)	38 (63)
	Upper-level hospital (300-500 beds)	15 (25)
	Primary care hospital (≤300 beds)	7 (12)
**Professional group, n (%)**		
	Physician	26 (43)
	ICU^a^ Nurse	10 (17)
	Medical student	24 (40)
**Work experience (years), n (%)**		
	<1	10 (17)
	1-3	4 (7)
	3-5	19 (32)
	5-10	11 (18)
	>10	16 (27)

^a^ICU: intensive care unit.

### Objective Parameters

The results of the analysis of the objective parameters—accuracy, time, and mental workload—showed that the participants performed best using the VIDS prototype. The exact results can be seen in Table 2. The differences between the systems were then analyzed using repeated measures ANOVA with a Greenhouse-Geisser correction.

**Table 2:** Descriptive statistics and objective parameters.

System	Accuracy, mean (SD)	Time (seconds), mean (SD)	RTLX^a^, mean (SD)
VIDS^b^	0.28 (0.52)	195.45 (79.08)	3.49 (1.52)
PDMS^c^	0.75 (0.88)	491.03 (177.63)	4.17 (1.62)
Paper	2.37 (1.46)	763.93 (242.14)	6.31 (1.71)

^a^RTLX: Raw Task Load Index.

^b^VIDS: voice information and documentation system.

^c^PDMS: patient data management system.

**Figure 3:**
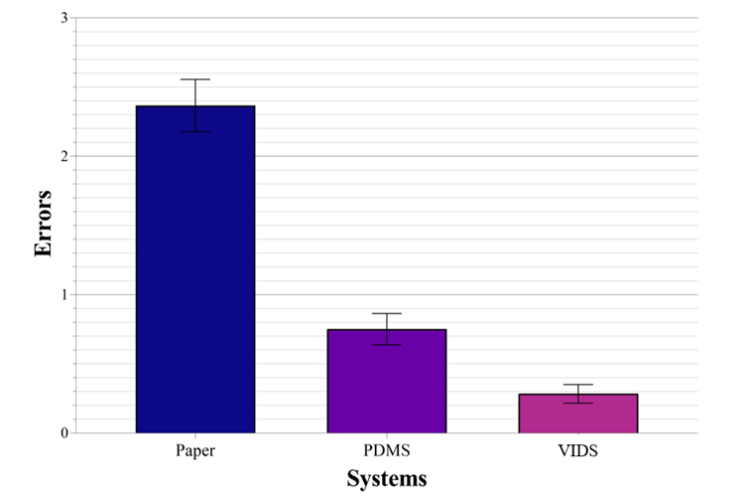
Mean errors with standard error of the mean. PDMS: patient data management system; VIDS: voice information and documentation system.

The analysis determined that there is a statistically significant difference in the errors made (n=60; F_1.73, 101.77_=78.92; η_p_^2^=0.57; *P*<.001). The statistically significant difference is visualized in Figure 3. The effect size is Cohen *d*=1.15 and thus shows a large effect [48]. Post hoc analysis with a Bonferroni adjustment revealed that by using the VIDS, significantly fewer errors were made compared to PDMS (1-tailed *t*_59_=3.45; Cohen *d*=0.45; *P*=.03) to paper-based documentation (*t*_59_=11.2; Cohen *d*=1.45; *P*<.001) [49]. Using PDMS, significantly fewer errors were made compared to paper-based documentation (*t*_59_=8.3; Cohen *d*=1.07; *P*<.001) [49].

**Figure 4:**
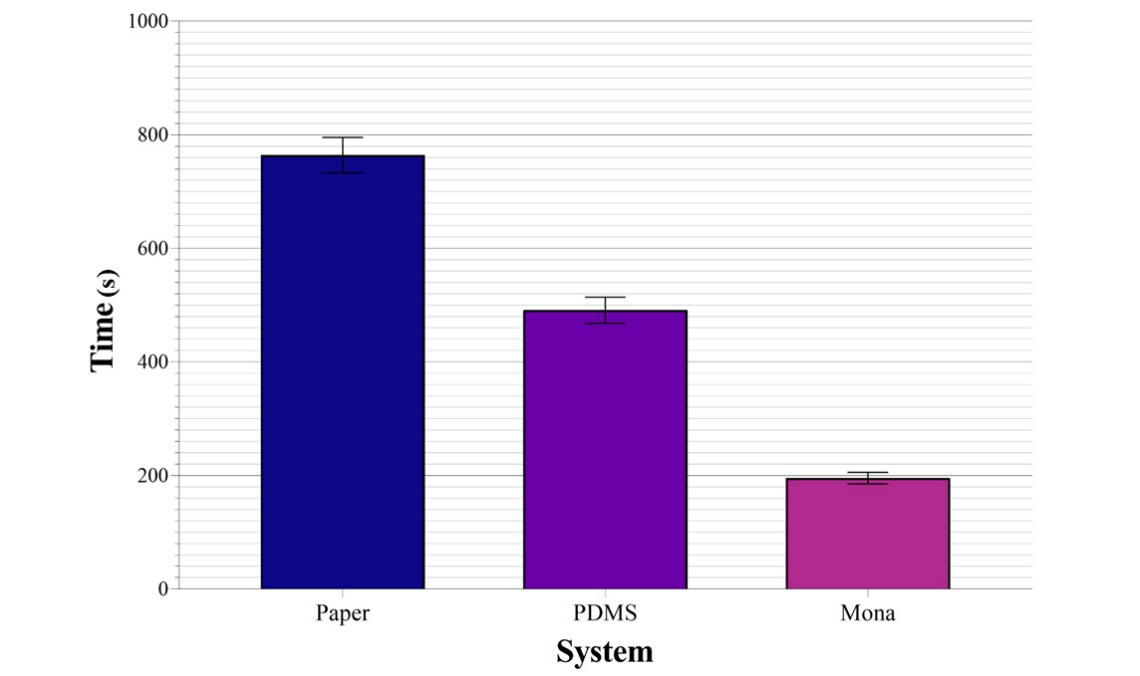
Mean measured time in between the study arms with standard error of the mean. PDMS: patient data management system; VIDS: voice information and documentation system.

Similar results were seen in the analysis of the time needed by the participants when completing the tasks, as can be seen in Figure 4. A statistically significant difference between the 3 systems was proven (n=60; F_1.65, 97.25_=188.84; η_p_^2^=0.76; Cohen *d*=1.79; *P*<.001) [48]. The post hoc analysis with Bonferroni adjustments confirmed that the use of the VIDS was statistically significantly faster (VIDS to PDMS: *t*_59_=12.48; Cohen *d*=1.61; *P*<.001; VIDS to paper: *t*_59_=20.41; Cohen *d*=2.63; *P*<.001; PDMS to paper: *t*_59_= 7.78; Cohen *d*=1; *P*<.001) [49].

**Figure 5:**
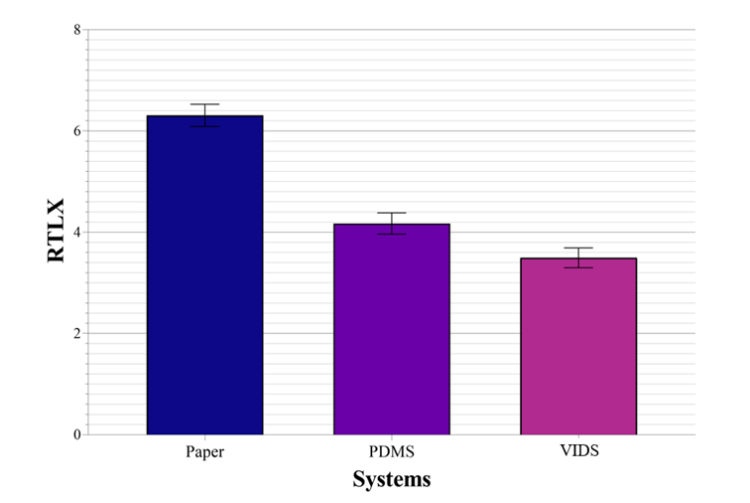
Raw Task Load Index (RTLX) with standard error of the mean. PDMS: patient data management system; VIDS: voice information and documentation system.

The repeated measures ANOVA of the mental workload also showed a statistically significant difference between the use of the 3 systems (n=60; F_1.82, 107.11_=56.91; η_p_^2^=0,49; Cohen *d*=0.98; *P*<.001), as shown in Figure 5 [48]. However, the post hoc analysis with Bonferroni adjustments only proved a statistically significant higher mental workload using paper-based documentation compared to PDMS (*t*_59_=9.27; Cohen *d*=1.2; *P*<.001) and VIDS (*t*_59_=9.16; Cohen *d*=1.18; *P*<.001) [49]. There is no statistically significant reduction in workload when using VIDS compared to PDMS (*t*_59_=2.4; *P*=.06) [49].

### Subjective User Experience

**Figure 6:**
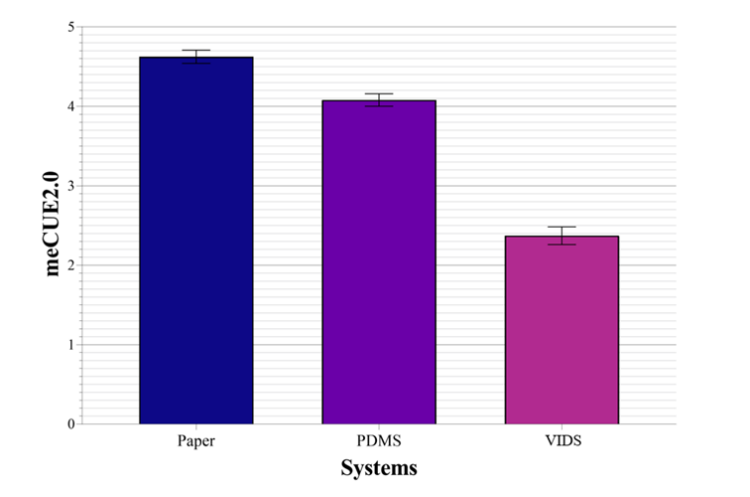
Mean meCUE2.0 score and standard error of the mean. PDMS: patient data management system; VIDS: voice information and documentation system.

In order to evaluate the subjective user perception, the answers of the meCUE2.0 were analyzed. The average meCUE2.0 score was 2.37 (SD 0.86) for paper documentation, 4.08 (SD 0.61) for PDMS, and 4.63 (SD 0.64) for VIDS (Figure 6). Thus, VIDS showed the highest user satisfaction, while the participants felt least satisfied with using paper-based documentation. The meCUE2.0 was further analyzed using the mean scores of each participant for each of the 3 systems. This assumption was tested using a repeated measures ANOVA followed by pairwise comparisons with Bonferroni correction. The ANOVA showed after Greenhouse-Geisser correction (Mauchly *W*=0.9; *P*=.04) a significant difference between the 3 tested systems (n=60; *F*_1.81, 106.78_=144.73; η_p_*^2^*=0.71; *P*<.001). The effect size is Cohen *d*=1.56, thus corresponding to a strong effect [48]. The pairwise comparisons with Bonferroni correction proved a statistically significant difference between all 3 systems with regard to user experience (*P*<.001) [49]. The effect size is Cohen *d*=0.61 for the comparison of VIDS with PDMS (*t*_59_=–4.7), Cohen *d*=1.87 for VIDS with paper documentation (*t*_59_=–14.5), and Cohen *d*=1.57 for PDMS with paper-based documentation (*t*_59_=–12.17). Consequently, the effect size always corresponds to a strong effect [48].

**Figure 7:**
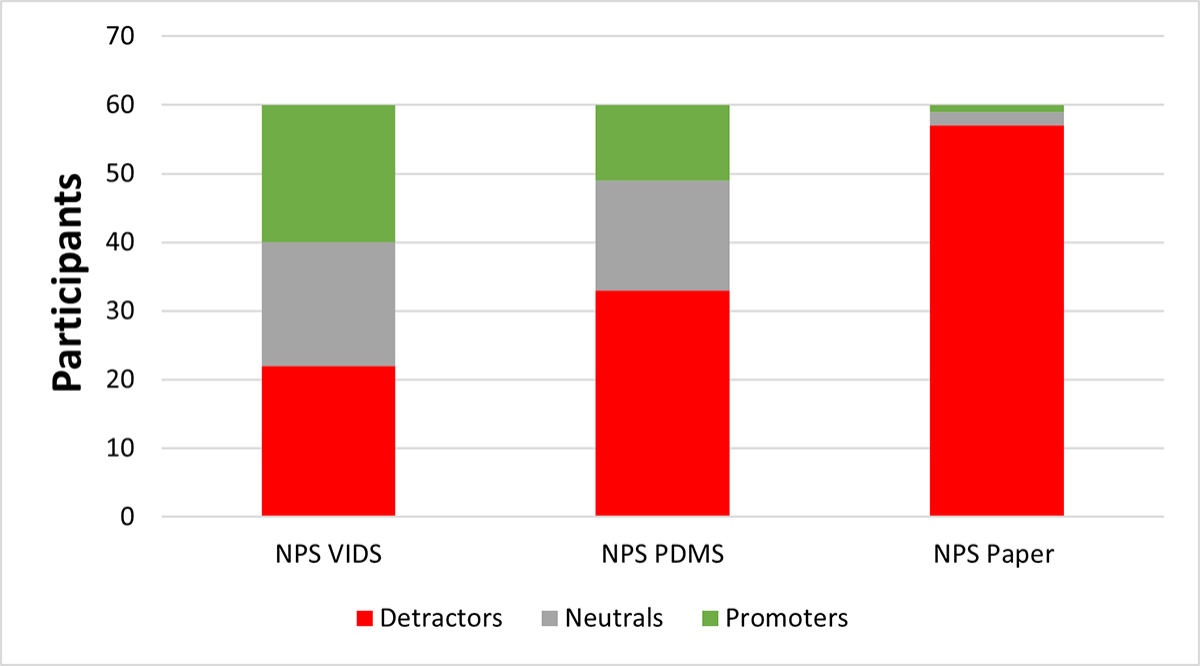
Distribution net promoter score (NPS). PDMS: patient data management system; VIDS: voice information and documentation system.

For each system, the NPS was calculated according to the given formula (NPS = promotors [% of all participants] minus detractors [% of all participants]) and compared as relative values. The NPS is a score correlating with actual consumer behavior and thus the ability of the company or system to be recommended [46]. The score is divided in promotors (score of 9 or higher), detractors (score of 6 or lower), and neutral users (score of 7-8). The distribution of the promotors and detractors can be seen in Figure 7. The comparison showed that the NPS for VIDS is 11.12 times higher than for PDMS and 28.27 times higher than for paper-based documentation (Figure 7).

The advantages of new, technologically advanced documentation systems could potentially be based on the technical affinity of the participants. In order to explore this assumption, we analyzed the effect of technical affinity on the results. The participants were grouped into high and low technical affinity groups using a median split according to the results of the TA-EG questionnaire. The analysis showed that the technical affinity has no statistically significant impact on the performance of the systems (Multimedia Appendices 4 and 5). Accordingly, it can be assumed that the prototype is beneficial regardless of the user’s affinity for technology.

## Discussion

### Overview

In reaction to the increasing workload in intensive care medicine and the growing ability to integrate AI applications into clinical routines, new technologies emerge, aiming to improve the treatment of patients with critical illnesses. This study was conducted in order to assess the performance, documentation accuracy, mental workload, and user experience associated with tasks typical of the ICU while using 2 established systems (paper-based documentation [PDMS] and an AI-based [VIDS]).

A total of 60 participants, consisting of physicians, nursing staff, and medical students, completed a set of defined tasks with each system. This was complemented by the completion of several questionnaires. With the time taken, the errors made, and the questionnaires, we compared the 3 systems objectively and based on the users’ subjective experiences. The results showed a statistically significant benefit in the objective variables of time and mistakes when using the VIDS. These advantages of the AI-based system are in line with several studies showing that speech recognition can reduce the documentation burden [32,34,35]. The results of this study should encourage the continuation of developing this and other AI-based software to reproduce these results in a real clinical setting.

Furthermore, not only the time needed to complete the tasks but also the errors made compared to the primarily established gold standard were significantly reduced when using the VIDS. This can be explained by the AI-based automated completion of the tasks and answers to the questions asked. Voice recognition in combination with AI reduces the number of necessary steps that the user has to take in order to correctly complete a task or answer a question. Therefore, fewer errors can be made. The significantly reduced error rate might also be due to the data presentation and user interface of the software. As Ahmed et al [17] showed in 2011, the presentation of data has a significant impact on the error rate.

Objective variables are important when evaluating the usefulness of a new system; however, subjective perception and workload are equally relevant. Comparing the RTLX results for each system showed that the VIDS and the PDMS were equivalent, as no statistically significant difference between these 2 systems could be observed. The graphical analysis of the data showed a discernible advantage of the AI-based system; however, this has not proven to be statistically significant. Because we had to limit the required time for study participation, the number of tasks to be completed within the study arms had to be limited as well. The VIDS used in the study was in prototypic stages and has potentially not yet shown its full strengths in terms of usability, consequently reducing mental workload. Therefore, future studies should elaborate on to what extent the further development of the VIDSs contributes to a change in this effect.

The questionnaires aiming to evaluate the subjective user experience confirmed that the participants felt an advantage when using the VIDS compared to PDMS and paper-based documentation (Multimedia Appendix 6). The meCUE 2.0 showed a statistically significantly higher score for the user experience compared to PDMS and paper documentation. As a high score represents a positive user experience, the results underline that new approaches are not only objectively but also subjectively advantageous in the defined tasks we tested.

These 2 analyses of the subjective user experience were filled out after the participants completed all tasks with every system. The results underline that the VIDS have not only shown equivalence and advantage, respectively, in the objective variables but also in the perceptions of the participants. This goes in line with a 2019 study by Momenipur et al [7] showing that physicians also feel constant time pressure while working and underlined the major importance to improve the subjective work experience and efficiency [7]. The results of the study suggest that by using voice-based software solutions, this can potentially be achieved. This is further underlined by the outcome of the perceived speed of documentation. A total of 80% (48/60) of the participants ranked the VIDS highest. Therefore, the work time was not only statistically significantly less when using the VIDS compared to PDMS and paper-based documentation but also subjectively the lowest. This is especially relevant, as Tajirian et al [50] showed in their study in 2020 that physicians tend to overestimate the time they spend with electronic health records.

### Limitations

Clearly, this study has some limitations. High efforts were undertaken in order to reduce confounders and circumstances between the study arms. However, as the 3 observed interventions are very different in technical requirements, input methods, and distribution among ICUs, the study can only be an indication for the acceptance and performance of these systems. Further studies in real-world ICUs are currently in preparation. These studies will analyze the accuracy, efficiency, and mental workload when using the new AI-based VIDS within an operating ICU. These studies will particularly analyze the roots of the observed effects (eg, which technical aspect contributes to what extent) in order to further direct development and progress in this field. Further, the analysis between different VIDSs and different user groups (eg, linguistic backgrounds and age) will be a closely analyzed. Furthermore, the study could not be performed in a real ICU setting due to infection constraints during the COVID-19 pandemic. Even though we tried to reproduce the high noise level of ICUs by performing the tests in a noisy simulation environment, studies in actual ICUs will have to be conducted in order to confirm the results. Another limitation of the study results from the tasks observed. The comparison of 3 different interaction methods, all developed in different decades, using very different technologies, and also requiring different training levels, is challenging and can only give an indication of the value of these systems in real-world health care usage. We attempted to overcome this by using standardization as much as possible and varying interventions in order to limit crossover effects. Additional studies are needed, taking in particular the technical details of VIDS and PDMS (eg, voice recognition, language understanding, data processing, and user interface) into account to make targeted development possible and address potential usability restrictions. As we only tested 3 aspects of the complex work on ICUs with predefined tasks, larger studies will have to confirm the benefits of the new software within the actual workflow of ICUs. Implementing complex IT systems, such as PDMS and VIDS, in a health care workflow certainly produces logistical and economic challenges, as new monitors and systems have to be installed during ongoing patient care in the ICU. Further, networking and IT infrastructure are required, and hospital prerequisites, such as the presence of digital patient data, have to be fulfilled. As documentation is an absolute necessity in patient care, this implementation might lead to disturbances and consequently increased workload, an effect that has to be taken closely into account.

Consequently, in order to assess the full picture of a system’s performance, it is required to consider the user’s personal perception as well as the objective measurements [50]. The well-being of the medical staff is closely connected to patient safety [10]. In the ranking of the highest user satisfaction, the VIDS was chosen most often on the first rank, as can be seen in Multimedia Appendix 7. This implies that using VIDS can potentially improve the quality of care and patient safety.

### Conclusions

A high standardization and objectification of the systems studied was one of the main goals of the study. Nevertheless, the diversity of the investigated systems, the different user interfaces, and the usage contexts inevitably create an inhomogeneity that cannot be completely eliminated. Therefore, the approach of this study was to choose a usage-centric and user-centered object of study. By choosing a diverse set, including novel and well-established metrics, we also tried to focus on the multidimensionality of the results. In order to enable the greatest possible comparability of the developed systems, the adoption of such a measurement method is essential.

Nevertheless, the published approach is only one of the possible solutions to the problem. At this point, we would like to explicitly encourage the use of the protocols we have developed and to further improve and objectify them. In the long term, only based on an established, manufacturer-independent protocol is it possible to approximate the comparison between the different approaches. As novel systems are arising, the proposed study protocol could be the starting point for the development of an industry-wide, vendor-independent accepted standard.

Overall, the results of this study confirm the potential of the use of AI in the clinical setting to reduce workload and improve patient care. The workload in ICUs is growing due to an increasing amount of data collected and the need to document, analyze, and interpret these data points for each patient. In conclusion, AI-based systems like VIDS have the potential to reduce this workload and improve evidence-based and safe patient care.

## Appendix

Multimedia Appendix 1 TA-EG table.

Multimedia Appendix 2 meCUE2.0 table.

Multimedia Appendix 3 Solution pathways for task completion. Details about solution pathways to all tasks with each system and necessary interaction with each system.

Multimedia Appendix 4 Technical affinity table.

Multimedia Appendix 5 Correlation technology affinity on performance.

Multimedia Appendix 6 User satisfaction table.

Multimedia Appendix 7 Working speed table.

## Abbreviations

AI: artificial intelligence
ICU: intensive care unit
NASA-TLX: National Aeronautics and Space Administration Task Load Index
NPS: net promoter score
PDMS: patient data management system
RTLX: Raw Task Load Index
TA-EG: Fragebogen zur Erfassung der Technikaffinität als Umgang mit und Einstellung zuelektronischen Geräte (“Questionnaire for the assessment of technology affinity as handling and attitude toward electronic devices”)
VIDS: voice information and documentation system

## References

[1] Mayrhofer O, Frey R, Mayrhofer O, Hügin W (1972). Definition, funktion und bedeutung der intensivmedizin. Lehrbuch der Anaesthesiologie, Reanimation und Intensivtherapie.

[2] Glas M, Pfortmüller C (2020). Mein Erster Dienst—Intensivmedizin.

[3] Flohr L, Beaudry S, Johnson KT, West N, Burns CM, Ansermino JM, Dumont GA, Wensley D, Skippen P, Gorges M (2018). Clinician-driven design of VitalPAD-an intelligent monitoring and communication device to improve patient safety in the intensive care unit. IEEE J Transl Eng Health Med.

[4] Butler R, Monsalve M, Thomas GW, Herman T, Segre AM, Polgreen PM, Suneja M (2018). Estimating time physicians and other health care workers spend with patients in an intensive care unit using a sensor network. Am J Med.

[5] Hefter Y, Madahar P, Eisen LA, Gong MN (2016). A time-motion study of ICU workflow and the impact of strain. Crit Care Med.

[6] Carayon P, Wetterneck TB, Alyousef B, Brown RL, Cartmill RS, McGuire K, Hoonakker PLT, Slagle J, Van Roy KS, Walker JM, Weinger MB, Xie A, Wood KE (2015). Impact of electronic health record technology on the work and workflow of physicians in the intensive care unit. Int J Med Inform.

[7] Momenipur A, Pennathur PR (2019). Balancing documentation and direct patient care activities: a study of a mature electronic health record system. Int J Ind Ergon.

[8] Wright AA, Katz IT (2018). Beyond burnout—redesigning care to restore meaning and sanity for physicians. N Engl J Med.

[9] Sinsky C, Colligan L, Li L, Prgomet M, Reynolds S, Goeders L, Westbrook J, Tutty M, Blike G (2016). Allocation of physician time in ambulatory practice: a time and motion study in 4 specialties. Ann Intern Med.

[10] Hall LH, Johnson J, Watt I, Tsipa A, O'Connor DB (2016). Healthcare staff wellbeing, burnout, and patient safety: a systematic review. PLoS One.

[11] Collins S, Couture B, Kang MJ, Dykes P, Schnock K, Knaplund C, Chang F, Cato K (2018). Quantifying and visualizing nursing flowsheet documentation burden in acute and critical care. AMIA Annu Symp Proc.

[12] Fischer JE, Calame A, Dettling AC, Zeier H, Fanconi S (2000). Experience and endocrine stress responses in neonatal and pediatric critical care nurses and physicians. Crit Care Med.

[13] Densen P (2011). Challenges and opportunities facing medical education. Trans Am Clin Climatol Assoc.

[14] Beecken WD, Matusiewicz D (2020). Changes—Analyse der Entwicklung der Digitalen Medizin im deutschen Gesundheitssystem aus ärztlicher Sicht. Think Tanks im Gesundheitswesen: Deutsche Denkfabriken und ihre Positionen zur Zukunft der Gesundheit.

[15] Martin L, Peine A (2021). [What is new… implementation of artificial intelligence in intensive care medicine: hype or already reality?]. Anaesthesist.

[16] Chao CA (2016). The impact of electronic health records on collaborative work routines: a narrative network analysis. Int J Med Inform.

[17] Ahmed A, Chandra S, Herasevich V, Gajic O, Pickering BW (2011). The effect of two different electronic health record user interfaces on intensive care provider task load, errors of cognition, and performance. Crit Care Med.

[18] Toll E (2020). The cost of technology. JAMA.

[19] Shanafelt TD, West CP, Sloan JA, Novotny PJ, Poland GA, Menaker R, Rummans TA, Dyrbye LN (2009). Career fit and burnout among academic faculty. Arch Intern Med.

[20] Grol R, Mokkink H, Smits A, van Eijk J, Beek M, Mesker P, Mesker-Niesten J (1985). Work satisfaction of general practitioners and the quality of patient care. Fam Pract.

[21] Manomenidis G, Panagopoulou E, Montgomery A (2019). Job burnout reduces hand hygiene compliance among nursing staff. J Patient Saf.

[22] Muinga N, Abejirinde IOO, Paton C, English M, Zweekhorst M (2021). Designing paper-based records to improve the quality of nursing documentation in hospitals: a scoping review. J Clin Nurs.

[23] Fröhlich D, Bittersohl C, Schroeder K, Schöttle D, Kowalinski E, Borgwardt S, Lang UE, Huber CG (2019). Reliability of paper-based routine documentation in psychiatric inpatient care and recommendations for further improvement. Front Psychiatry.

[24] Castellanos I, Ganslandt T, Prokosch HU, Schüttler J, Bürkle T (2013). [Implementation of a patient data management system. Effects on intensive care documentation]. Anaesthesist.

[25] Ballermann MA, Shaw NT, Arbeau KJ, Mayes DC, Gibney RTN (2010). Impact of a critical care clinical information system on interruption rates during intensive care nurse and physician documentation tasks. Stud Health Technol Inform.

[26] Cheung A, van Velden FHP, Lagerburg V, Minderman N (2015). The organizational and clinical impact of integrating bedside equipment to an information system: a systematic literature review of Patient Data Management Systems (PDMS). Int J Med Inform.

[27] Baumann LA, Baker J, Elshaug AG (2018). The impact of electronic health record systems on clinical documentation times: a systematic review. Health Policy.

[28] Hodgson T, Magrabi F, Coiera E (2017). Efficiency and safety of speech recognition for documentation in the electronic health record. J Am Med Inform Assoc.

[29] Esteva A, Robicquet A, Ramsundar B, Kuleshov V, DePristo M, Chou K, Cui C, Corrado G, Thrun S, Dean J (2019). A guide to deep learning in healthcare. Nat Med.

[30] Dymek C, Kim B, Melton GB, Payne TH, Singh H, Hsiao CJ (2021). Building the evidence-base to reduce electronic health record-related clinician burden. J Am Med Inform Assoc.

[31] Hakes B, Whittington J (2008). Assessing the impact of an electronic medical record on nurse documentation time. Comput Inform Nurs.

[32] Goss FR, Blackley SV, Ortega CA, Kowalski LT, Landman AB, Lin CT, Meteer M, Bakes S, Gradwohl SC, Bates DW, Zhou L (2019). A clinician survey of using speech recognition for clinical documentation in the electronic health record. Int J Med Inform.

[33] Zuchowski M (2020). Medizinische spracherkennung: weniger dokumentationsaufwand, mehr zeit: seit einigen jahren wird die medizinische spracherkennung regelmäßig zur unterstützung der dokumentation in krankenhäusern eingesetzt. Dies beeinflusst die interdisziplinäre zusammenarbeit und spart zeit, die stattdessen in patientennahe tätigkeiten einfließen kann. Ihr volles Potenzial kann medizinische Spracherkennung allerdings erst entfalten, wenn sie mit anderen Anwendungen vernetzt ist. KMA.

[34] Blackley SV, Schubert VD, Goss FR, Al Assad W, Garabedian PM, Zhou L (2020). Physician use of speech recognition versus typing in clinical documentation: a controlled observational study. Int J Med Inform.

[35] Hodgson T, Coiera E (2016). Risks and benefits of speech recognition for clinical documentation: a systematic review. J Am Med Inform Assoc.

[36] Licht A, Blaser J (2002). [Speech recognition in clinical routine, a pilot trial at the Zurich University Hospital]. Praxis (Bern 1994).

[37] Poncette AS, Spies C, Mosch L, Schieler M, Weber-Carstens S, Krampe H, Balzer F (2019). Clinical requirements of future patient monitoring in the intensive care unit: qualitative study. JMIR Med Inform.

[38] Cosgriff CV, Celi LA, Stone DJ (2019). Critical care, critical data. Biomed Eng Comput Biol.

[39] (2015). IntelliSpace critical care and anesthesia: release H technical data sheet. Koninklijke Philips N.V.

[40] Consortium Uniklinik RWTH Aachen, RWTH Aachen, SERMAS, CapDigital, ATOS, Hochschule Trier (2021). DEL05 final report: 20655—clinical artificial intelligence improving healthcare. Claire, The Virtual Healthcare Assistant.

[41] Karrer K, Glaser C, Clemens C, Bruder C (2009). Berliner werkstatt mensch-maschine-systeme. Beiträge 8.

[42] Hart SG (2006). Nasa-Task Load Index (NASA-TLX); 20 years later. Proc Hum Factors Ergon Soc Annu Meet.

[43] Hendy KC, Hamilton KM, Landry LN (1993). Measuring subjective workload: when is one scale better than many?. Hum Factors.

[44] Mital A (1989). Advances in Industrial Ergonomics and Safety: v. 1 (Proceedings of the Annual International Industrial Ergonomics & Safety Conference held in Cincinnati, Ohio, USA, 5-9 June 1989).

[45] Minge M (2018). Nutzererleben messen mit dem meCUE 2.0—Ein Tool für alle Fälle?.

[46] Reichheld FF (2003). The one number you need to grow. Harv Bus Rev.

[47] Eid M, Gollwitzer M, Schmitt M (2015). Statistik und Forschungsmethoden: Lehrbuch. Mit Online-Material.

[48] Cohen J (2013). Statistical Power Analysis for the Behavioral Sciences.

[49] Bland JM, Altman DG (1995). Multiple significance tests: the Bonferroni method. BMJ.

[50] Tajirian T, Stergiopoulos V, Strudwick G, Sequeira L, Sanches M, Kemp J, Ramamoorthi K, Zhang T, Jankowicz D (2020). The influence of electronic health record use on physician burnout: cross-sectional survey. J Med Internet Res.

