# Supplementary material for: Standardized Comparison of Voice-Based Information and Documentation Systems to Established Systems in Intensive Care: Crossover Study
Source: JMIR Med Inform. 2023 Nov 28;11:e44773. doi: 10.2196/44773 (PMC10716746; doi:10.2196/44773)
Supplement: Multimedia Appendix 1 [file medinform_v11i1e44773_app1.docx]

**Table S1.** TA-EG.

| **Scale** | **Items** (Five-point Likert-Scale: “Fully applies”; “Partly applies”; “Partly/partly”; “Rather not applies”; “Does not apply”) |
| --- | --- |
| Enthusiasm | I inform myself about electronic devices, even if I have no intention of buying them.  I love having new electronic devices.  I get excited when a new electronic device is introduced to the market.  I enjoy trying out an electronic device. |
| Expertise | I know most of the functions of the electronic devices I own.  I have or would have problems reading electronic/computer magazines.  I find it easy to learn how to use electronic devices.  I know my way around electronic devices. |
| Positive Stance | Electronic devices help to get information.  Electronic devices enable a high standard of living.  Electronic devices increase security.  Electronic devices make you independent.  Electronic devices make my everyday life easier. |
| Negative Stance | Electronic devices make people ill.  Electronic devices lead to mental impoverishment.  Electronic devices make many things more cumbersome.  Electronic devices reduce personal contact between people.  Electronic devices cause stress. |
