# Supplementary material for: Standardized Comparison of Voice-Based Information and Documentation Systems to Established Systems in Intensive Care: Crossover Study
Source: JMIR Med Inform. 2023 Nov 28;11:e44773. doi: 10.2196/44773 (PMC10716746; doi:10.2196/44773)
Supplement: Multimedia Appendix 2 [file medinform_v11i1e44773_app2.docx]

**Table S1.** meCUE2.0.

| **System** | **Items** (Six-point Likert-Scale “I completely refuse”; “I refuse”; “I rather refuse”; “I rather agree”; “I agree”; “I completely agree”) |
| --- | --- |
| Paper | 1. The functions of the system are exactly right for my goals. 2. It quickly becomes clear how to use the system. 3. I find the system absolutely useful for use in intensive care units. 4. The operation of the system is understandable. 5. The system helps me to achieve my goals in the ICU. 6. The system frustrates me. 7. Compared to this system, other systems seem imperfect. 8. I would reintroduce exactly this system in intensive care units. |
| PDMS | 1. The functions of the system are exactly right for my goals. 2. It quickly becomes clear how to use the system. 3. I find the system absolutely useful for use in intensive care units. 4. The operation of the system is understandable. 5. The system helps me to achieve my goals in the ICU. 6. The system frustrates me. 7. Compared to this system, other systems seem imperfect. 8. I would reintroduce exactly this system in intensive care units. |
| VIDS | 1. The functions of the system are exactly right for my goals. 2. It quickly becomes clear how to use the system. 3. I find the system absolutely useful for use in intensive care units. 4. The operation of the system is understandable. 5. The system helps me to achieve my goals in the ICU. 6. The system frustrates me. 7. Compared to this system, other systems seem imperfect. 8. I would reintroduce exactly this system in intensive care units. |
