# Supplementary material for: Standardized Comparison of Voice-Based Information and Documentation Systems to Established Systems in Intensive Care: Crossover Study
Source: JMIR Med Inform. 2023 Nov 28;11:e44773. doi: 10.2196/44773 (PMC10716746; doi:10.2196/44773)
Supplement: Multimedia Appendix 3 [file medinform_v11i1e44773_app3.docx]

**Table S1.** Solution pathways for task-completion. Details about solution pathways to all tasks with each system and necessary interaction with each system.

| **Task Category** | **Task** | **Solution Pathway VIDS** | **Solution Pathway PDMS** | **Solution Pathway Paper** |
| --- | --- | --- | --- | --- |
| **Documentation** | - “Document 300mg amiodarone i.v. now”  - “Document 1.5g piperacillin/tazobactam (Tazobac) intravenously now/at 10.00 am”  - “Document 20mg furosemide now” | 1. Activate System (“Hey Mona”) 2. Give voice command 3. Wait for answer 4. Check correctness of answer 5. Task complete | 1. Choose tab “Prescriptions” on the left 2. Choose “Medication” in 1. 3. Choose correct medication in 2. 4. Add correct information for prescription according to task in 3. 5. Press OK | 1. Find list of documented medications 2. Write down medication and instructions in a new row 3. Sign order at the end of the row |
|  | - “Document the administration of a red blood cell concentrate / fresh frozen plasma at 1:00 a.m. for procedures numbered 1101002233 and indication active bleeding” | 1. Activate System (“Hey Mona”) 2. Give voice command 3. Wait for answer 4. Check correctness of answer 5. Task complete | 1. Choose tab “Prescriptions” on the left 2. Choose “Blood Products” in 1. 3. Choose correct blood product in 3. 4. Add correct information for blood product (Charge number, indication) according to task in 3. 5. Press OK | 1. Find page to document measures 2. Handwrite application of blood product with time and indication 3. Sign documentation |
| **Discovery of patient status** | - “What was the lactate trend in the last 12 hours?”  - “How did the creatinine levels develop within the last 4 days?” | 1. Active system (“Hey Mona”) 2. Ask to show the lab values 3. See lab values 4. Describe trend | 1. Choose tab “Lab” on the left 2. Find the correct row for the lab value asked for 3. Choose the correct time frame to answer question (top right corner of lab value table) 4. Describe trend | 1. Find printed out lab values in file 2. Find row with lab values asked for and correct date 3. Describe trend |
| **Score generation** | “What is the patient's current SOFA score?” | 1. Activate system (“Hey Mona”) 2. Ask to see patient summery 3. See current SOFA-Score | 1. Choose tab “Scores” on the left 2. Select SOFA in the row of Scores 3. See most recent SOFA Score | 1. Look at table (help how to calculate SOFA-Score) 2. Find each necessary value in file 3. Find Score of each value 4. Calculate SOFA Score (Calculator was given) |
