# Supplementary material for: Standardized Comparison of Voice-Based Information and Documentation Systems to Established Systems in Intensive Care: Crossover Study
Source: JMIR Med Inform. 2023 Nov 28;11:e44773. doi: 10.2196/44773 (PMC10716746; doi:10.2196/44773)
Supplement: Multimedia Appendix 4 [file medinform_v11i1e44773_app4.docx]

**Table S1.** Technical affinity.

| **Variable** | **Value** |
| --- | --- |
| Total Number of Participants | n=60 |
| **Technical Affinity** (n, %)  High  Low | 28 (46.67)  32 (53.33) |
| **Interaction System with Technical Affinity** (F, df, P) tested with Mixed ANOVA after median split  Time  Errors  RTLX | 1.06 (1.66, 96.28), 0.341  0.58 (1.73, 100.44), 0.539  0.28 (2, 116), 0.756 |
