# Supplementary figures and images for: Standardized Comparison of Voice-Based Information and Documentation Systems to Established Systems in Intensive Care: Crossover Study
Source: JMIR Med Inform. 2023 Nov 28;11:e44773. doi: 10.2196/44773 (PMC10716746; doi:10.2196/44773)

**Figure S1.** Correlation technology affinity on performance.

Monaona

Monaona

Monaona

Monaona


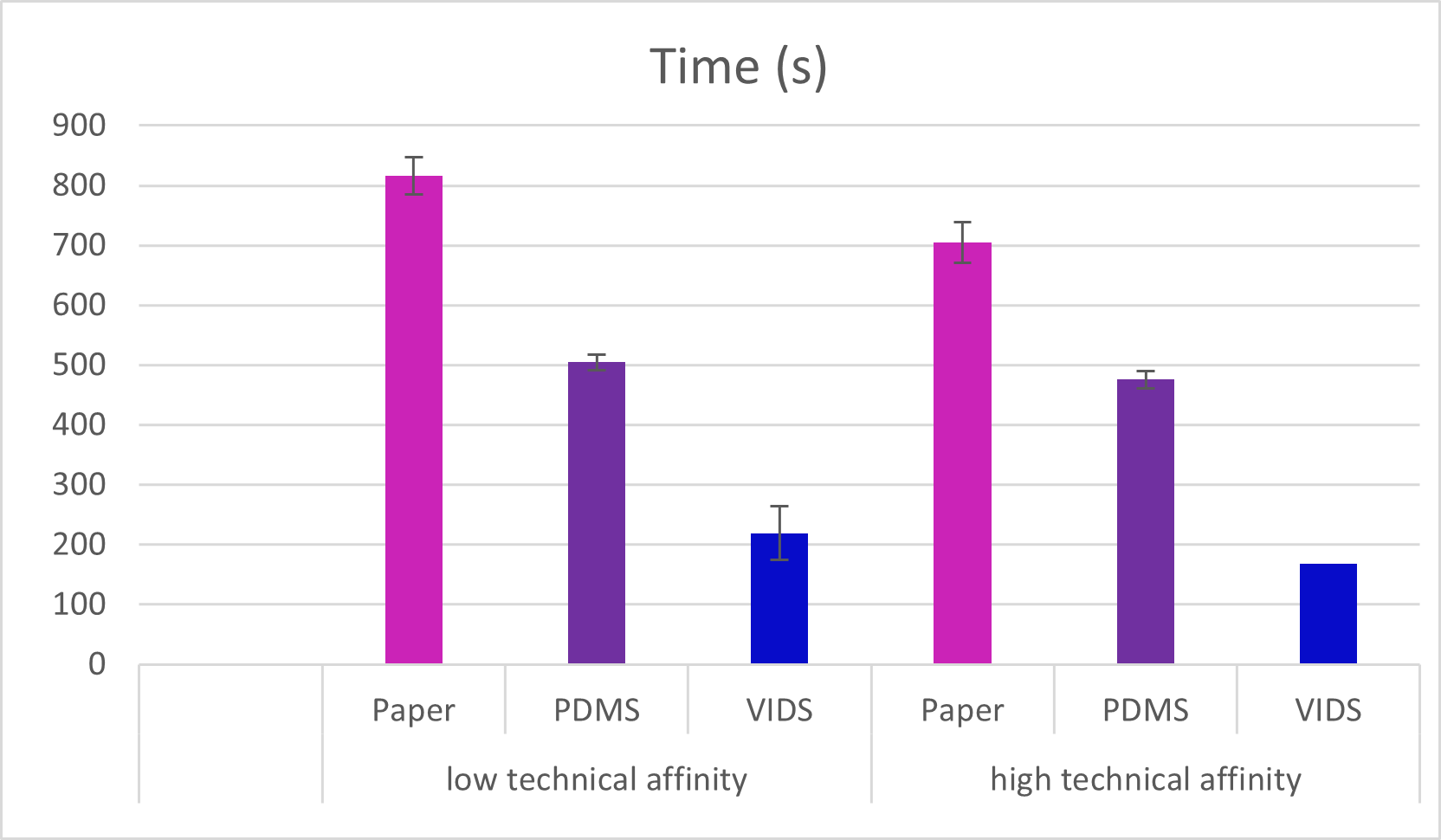


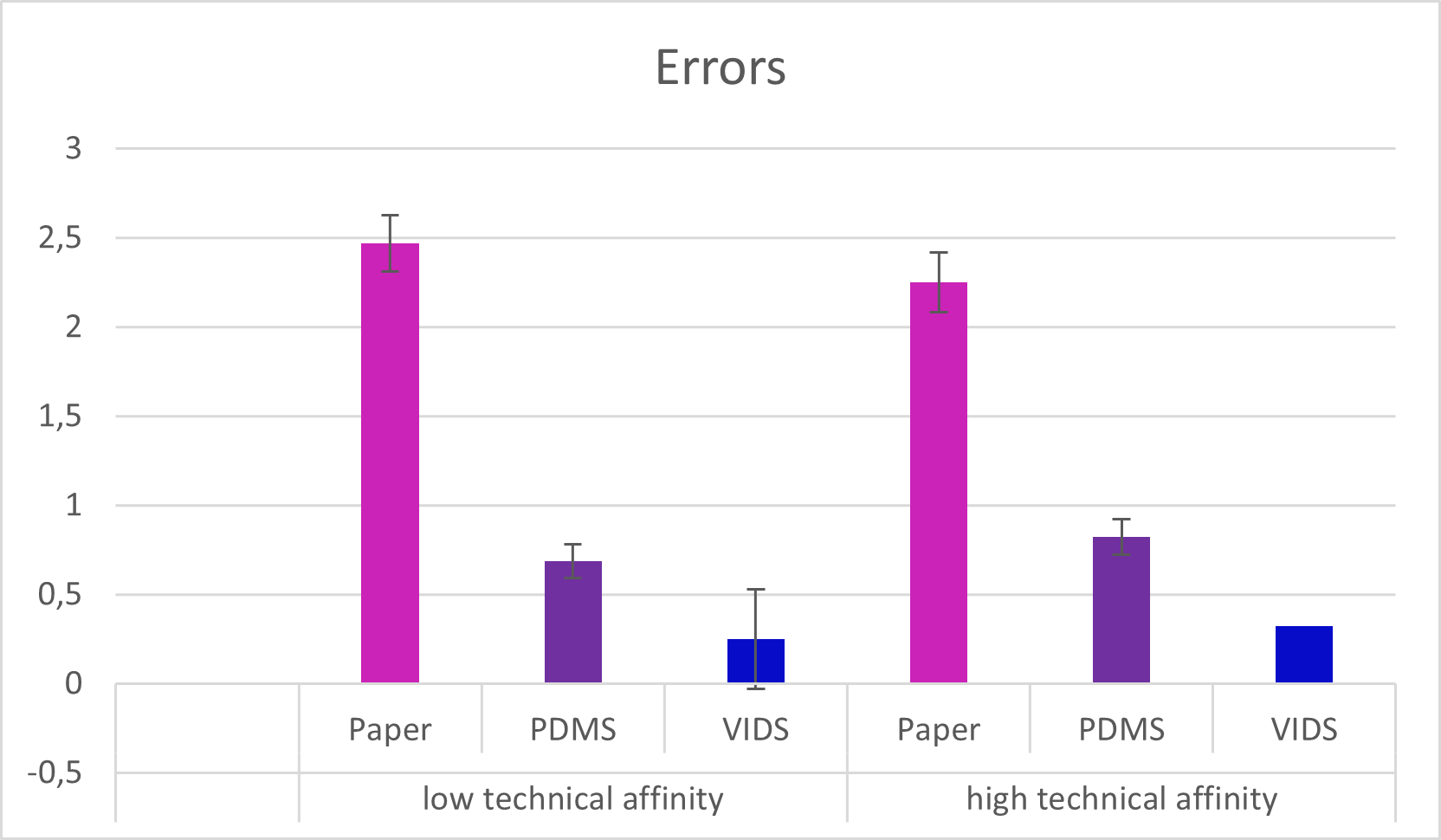


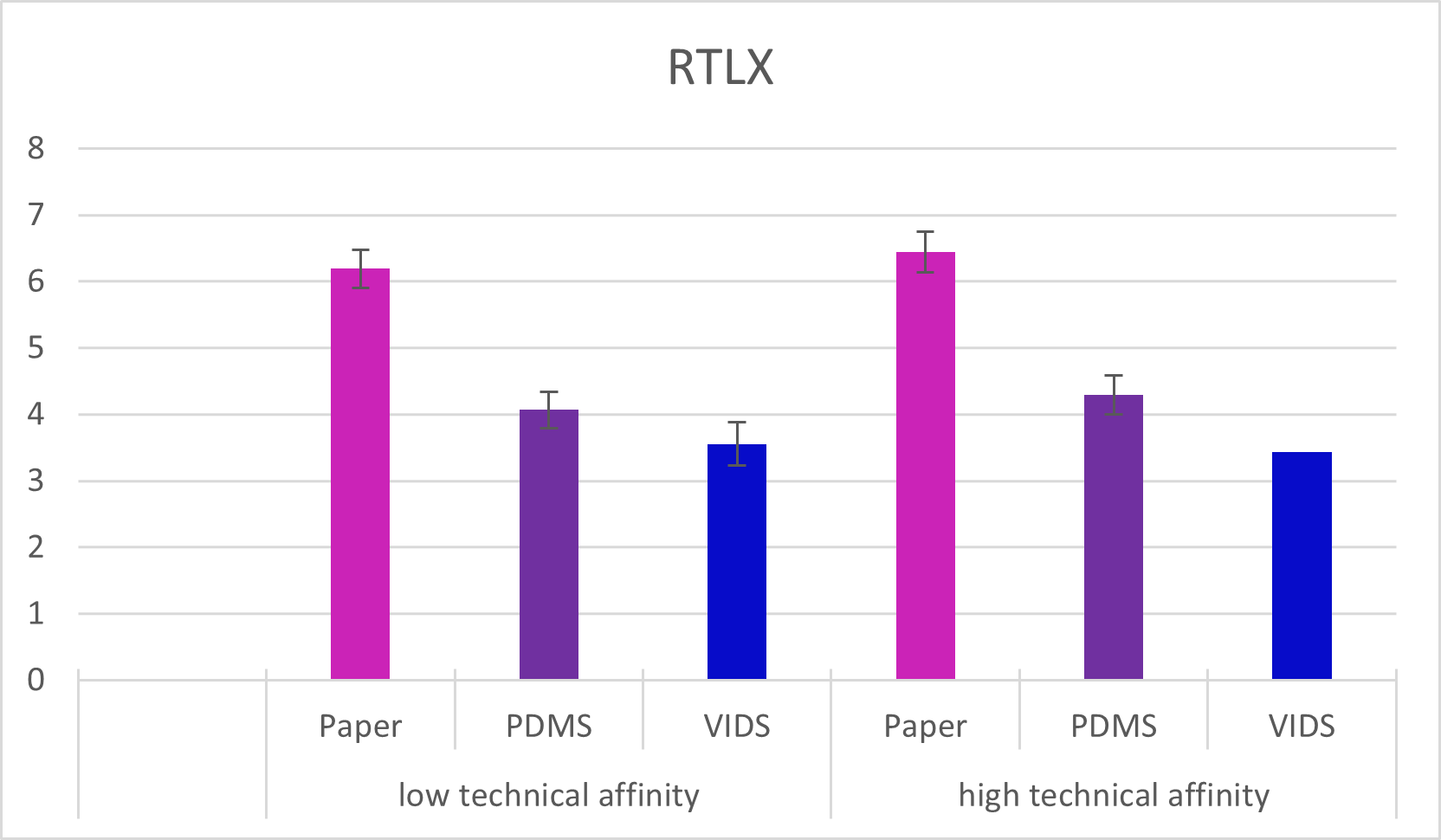

Supplement: Multimedia Appendix 5 [file medinform_v11i1e44773_app5.docx]

**Figure S1.** Working speed.


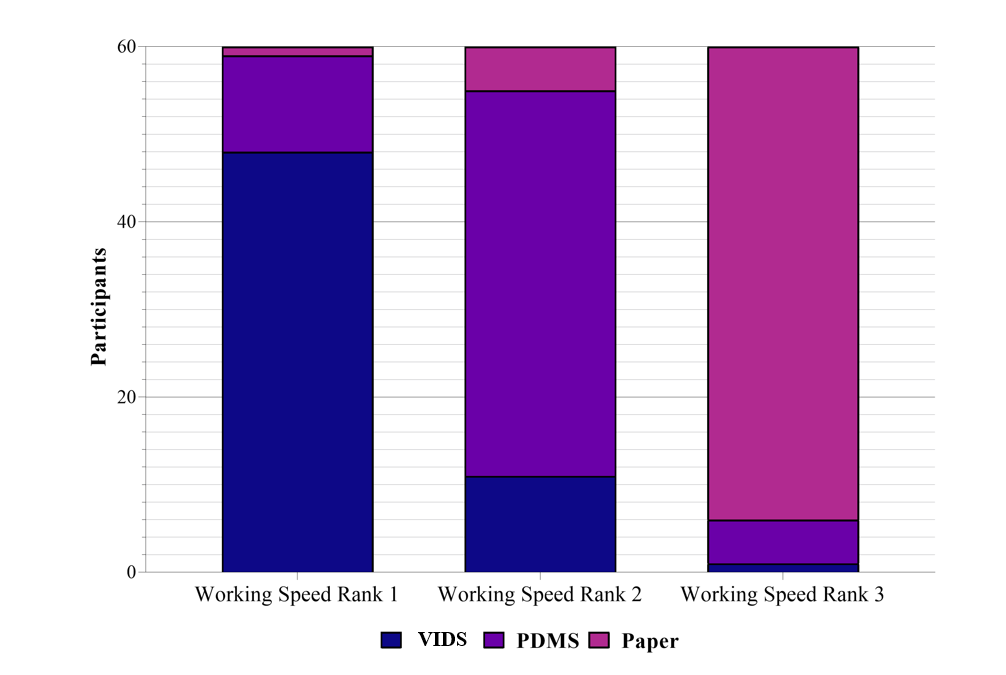

Supplement: Multimedia Appendix 7 [file medinform_v11i1e44773_app7.docx]
