# Supplementary material for: Standardized Comparison of Voice-Based Information and Documentation Systems to Established Systems in Intensive Care: Crossover Study
Source: JMIR Med Inform. 2023 Nov 28;11:e44773. doi: 10.2196/44773 (PMC10716746; doi:10.2196/44773)
Supplement: Multimedia Appendix 6 [file medinform_v11i1e44773_app6.docx]

**Table S1.** User satisfaction.

| **User Satisfaction Rank 1** | | | | |
| --- | --- | --- | --- | --- |
|  | | Frequency | Percent |  |
| VIDS | 30 | | 50,0 |  |
| PDMS | 29 | | 48,3 |  |
| ICCA | 1 | | 1,7 |  |
| Total | 60 | | 100,0 |  |
